# Open tibia/fibula in the elderly: A retrospective cohort study

**DOI:** 10.1016/j.jpra.2021.09.003

**Published:** 2021-10-01

**Authors:** Alice Lee, Luke Geoghegan, Grant Nolan, Kerri Cooper, Jonathan Super, Michael Pearse, Satyajit Naique, Shehan Hettiaratchy, Abhilash Jain

**Affiliations:** a Department of Plastic and Reconstructive Surgery, St. Mary's Hospital, Imperial College Healthcare NHS Trust, London, UK; b Department of Orthopaedic Surgery, St. Mary's Hospital, Imperial College Healthcare NHS Trust, London, UK

**Keywords:** ankle fracture, tibial fracture, lower limb fracture, elderly, trauma, free flap reconstruction, open fracture, limb reconstruction, limb salvage

## Abstract

The incidence of open tibia/fibula fractures in the elderly is increasing, but current national guidelines focus on the aggressive treatment of high-energy injuries in younger patients. There is conflicting evidence regarding whether older age affects treatment provision and outcomes in open fractures. The aim of this study was to determine if elderly patients are sustaining a different injury to younger patients and how their treatment and outcomes differ. This may have implications for future guidelines and verify their application in the elderly.

In this retrospective single centre cohort study (December 2015–July 2018), we compared the injury characteristics, operative management and outcomes of elderly (≥65 years) and younger (18–65 years) patients with open tibia/fibula fractures. An extended cohort examined free flap reconstruction.

In total, 157 patients were included. High-energy injuries were commoner in younger patients (88% vs 37%; p<0.001). Most were Gustilo-Anderson IIIb in both age groups. Elderly patients waited longer until debridement (21:19 vs 19:00 h) and had longer inpatient stays (23 vs 15 days). There was no difference in time to antibiotics, operative approach or post-operative complications.

Despite the low-energy nature of elderly patients’ injuries, the severity of soft tissue insult was equivalent to younger patients with high-energy injuries. Our data suggest that age and co-morbidities should not prohibit lower limb reconstruction. The current application of generic guidelines appears suitable in the elderly, particularly in the acute management. We suggest current management pathways and targets be reviewed to reflect the greater need for peri-operative optimisation and rehabilitation in elderly patients.

## Introduction

Open fractures are injuries whereby the fractured bone is exposed to the external environment through traumatised soft tissue, predisposing to infection.^1^ Open tibial diaphyseal fractures are the commonest open long bone fractures.^2^ Open tibia/fibula fractures predominantly occur in a bimodal age distribution with young males sustaining high-energy traumatic injuries and elderly females sustaining low-energy fragility fractures.^2^

The joint British Association of Plastic, Reconstructive and Aesthetic Surgeons (BAPRAS) and British Orthopaedic Association Standards for Trauma and Orthopaedics (BOAST) guideline for open fractures comprises a series of evidence-based standards for national practice.^3^ Key targets include the provision of prophylactic intravenous antibiotics within 1 h of injury, debridement within 12 or 24 h for high- and low-energy mechanisms, respectively, and definitive soft tissue coverage within 72 h. These guidelines appear focussed on the aggressive management of high-energy injuries in the otherwise fit and well, and outcomes from open fractures have dramatically improved over time.^4^

Over the past decade, there has been a significant demographic shift in patients with major traumatic injuries, with a greater proportion of patients aged ≥65 years sustaining high severity injuries. Elderly patients with open tibia/fibula fractures appear to have lower energy mechanisms compared with younger patients, such as falls from <2 m.^5^ There is conflicting evidence regarding the treatment and outcomes of open tibia/fibula fractures in the elderly compared with younger patients. Previous research has indicated worse outcomes in elderly patients, with respect to compliance with national standards (time to major trauma centre and initiation of antibiotics)^6^ and increased morbidity and mortality.^7^, ^8^, ^9^, ^10^, ^11^ On the contrary, Ovaska et al. reported that patients with open tibia/fibula fractures with complications (chronic pain, reoperations and clinic visits) were significantly younger than those without complications, possibly due to higher rates of high-energy trauma in this group.^12^ Some authors have suggested that age is less important than injury characteristics and associated injuries for predicting in-hospital mortality^9^ and complications.^13^ Indeed, Wijendra et al. demonstrated that elderly patients did not have significantly more complications than younger patients with similar injuries, and that outcomes were improved with early definitive fixation and primary wound closure.^13^ Many other injuries share a similar bimodal distribution, however the treatment strategies for the young and elderly are often distinct (e.g., neck of femur fractures, where young adults sustaining this high-energy injury are operated on emergently, with the aim of preserving the native hip joint^14^).

At present, open fractures in any age are managed under the single BOAST guidelines which seem counterintuitive. The aim of this study was to determine if the treatment and outcome in elderly patients sustaining open tibia/fibula fractures are different to younger patients, to help inform future guidelines and to clarify if their application in this elderly age group is justified.

## Materials and methods

A retrospective cohort study was performed on consecutive patients with open tibia/fibula fractures presenting to a single major trauma centre (MTC) between December 2015 to July 2018, with a minimum follow up of 1 year*.* We focussed on limb salvage and reconstruction, so patients were excluded if they presented with a limb amputation or died in the hospital before definitive limb reconstruction. Patients were also excluded if they were transferred or referred from another hospital as it was not possible to establish their initial treatment reliably, or if they had no documented follow up.

Data collection included patient demographics (age, gender and co-morbidities), injury characteristics (high vs low energy, Gustilo-Anderson grading, fracture type), operative management, compliance with BOAST guidelines and treatment outcomes (non-union, amputation, deep bone infection and time to discharge). Patients were defined as ‘elderly’ if they were ≥65 years at the time of injury, as has been reported previously.^6^ Comorbidities included diabetes, immunosuppression due to disease or medications and peripheral vascular disease. Immunosuppressed status was assessed using patients’ medical and drug history, in line with previous literature.^15^ High-energy injury was defined as a fall >2 m or collisions with a moving vehicle (motorcycle, scooter, car, bus, heavy goods vehicle, train) and low-energy was defined as a fall from <2 m. Non-union was defined as a complete cessation of the reparative processes of bone healing.^16^ As the initial cohort study yielded a low number of free flap reconstructions for our institution,^17^^,^^18^ to specifically investigate whether elderly patients are suitable for lower limb free flap reconstruction, an extended cohort of all patients undergoing this type of reconstruction was undertaken on consecutive patients between February 2012 to August 2019.

### Statistical analysis and study reporting

Group differences in continuous variables were evaluated using Student's t-test or Mann–Whitney U test depending on data distribution. Group differences in nominal data were analysed using Pearson's chi-square or Fisher's exact test. Significance was set at the level of P ≤0.05. Statistical analysis was carried out in SPSS version 26 (IBM, USA). This study is reported in line with the STROBE guideline.^19^

## Results

In total, 157 patients were included (See Table 1). Patients were excluded (n=61) for the following reasons: death before discharge (n=2), transfer or referral from another hospital (n=33), aged <18 years (n=1), lost to follow-up after discharge (n=16) and insufficient data (n=9).

**Table 1:** Patient demographics and comorbidities by age group.

	Aged <65 years	Aged ≥65 years
**Gender**		
Female	27 (21%)	19 (66%)
Male	101 (79%)	10 (34%)
**Comorbidities**		
Smoking*	45 (35%)	1 (3%)
Diabetes mellitus	6 (5%)	6 (21%)
Immunosuppression	0 (0%)	1 (3%)
Peripheral vascular disease	0 (0%)	1 (3%)
Other	33 (28%)	15 (52%)
None	86 (67%)	3 (10%)

⁎ Missing data in n=47.

### Demographic details

Most included patients were aged <65 years (128/157, 82%). Smoking was significantly more common in the under-65s (35%, 45/128) compared to the elderly cohort (3%, 1/29); p<0.001. The presence of medical co-morbidities was significantly more common in the elderly (p<0.001).

### Injury characteristics

A significantly greater proportion of injuries in the <65 years cohort was classified as high-energy compared with the ≥65 years cohort (88% vs 37%; p<0.001). For both age groups, most injuries were Gustilo-Anderson grade IIIb and involved both the tibia and fibula. There was no significant difference in Gustilo-Anderson classification (p=0.33) or bones fractured (p=0.99) between age groups (Table 2).

**Table 2:** Injury details and operative management by age group.

	Aged <65 years	Aged ≥65 years
**Injury mechanism**^a^		
High	106 (88%)	10 (37%)
Low	15 (12%)	17 (63%)
**Bones broken**		
Fibula only	8 (6%)	1 (3%)
Tibia only	32 (25%)	5 (17%)
Fibula and tibia	88 (69%)	23 (80%)
**Gustilo-Anderson grade**^b^		
I	7 (6%)	1 (4%)
II	29 (25%)	4 (16%)
IIIa	10 (9%)	1 (4%)
IIIb	60 (53%)	19 (76%)
IIIc	8 (7%)	0 (0%)
**Definitive osseous fixation modality**^c^		
Cast/boot	8 (7%)	1 (4%)
IM nail	37 (30%)	7 (26%)
External fixation	45 (37%)	11 (41%)
ORIF	28 (23%)	8 (30%)
Other	5 (4%)	0 (0%)
**Soft tissue coverage modality**^d^		
Direct closure	75 (66%)	12 (43%)
Free flap	11 (10%)	2 (7%)
Local or regional flap	20 (18%)	6 (21%)
SSG	7 (6%)	7 (25%)
VAC	1 (1%)	1 (4%)

Abbreviations: IM, intramedullary; ORIF, open reduction internal fixation; SSG, split thickness skin graft; VAC, vacuum assisted closure.

a = missing data for n=9 patients

b = missing data for n=18 patients

c = missing data for n=7 patients

d = missing data for n=15 patients.

### Operative management

There was no difference in the method of definitive bone fixation or soft tissue coverage between age groups (see Table 2). During the study period, thirteen patients underwent free flap reconstruction, including 11 patients aged 18–65 and 2 patients aged ≥65 years. Due to low numbers, this was further investigated in the extended free flap cohort (see below).

### Time to antibiotics and debridement

Time to antibiotic administration was not significantly longer for elderly patients compared with under-65’s (median time 2 h 47 min vs. 1 h 59 min, respectively, p= 0.081). Time to debridement for the elderly was longer than those aged <65 years (median time 21 h 19 min vs. 19 h, respectively; p=0.019). There was no difference between age groups with respect to compliance with BOAST4 targets for antibiotic administration, debridement and soft tissue coverage (Table 3).

**Table 3:** Compliance with BOAST4 criteria stratified by age.

BOAST4 standard	< 65 compliance, n (%); n=128	≥ 65 compliance, n (%); n=29	P-value
**Antibiotics within 1 h**^a^	11/92 (12%)	2/18 (11%)	0.92
**Debridement within 12 h for high energy injuries**^b^	22/71 (31%)	1/8 (13%)	0.47
**Debridement within 24 h for low energy injuries**^c^	8/13 (62%)	5/13 (38%)	0.24
**Soft tissue coverage by 72 h**^d^	67/103 (65%)	10/22 (45%)	0.09

N numbers are presented as fractions, where the numerator is the number of patients meeting BOAST4 standards and the denominator is the total group size.

a Missing data for n=36 for patients aged <65 and n=11 for patients aged ≥65 years.

b Missing data for n=35 patients aged <65 years and n=2 patients aged ≥65 years.

c Missing data for n=2 for patients aged <65 years and n=4 patients aged ≥65 years.

d Missing data for n=25 patients aged <65 years and n=7 patients aged ≥65 years.

### Outcomes

Time to discharge was longer in the elderly compared with the under-65s (median time 23 vs 15 days, respectively; p=0.03). There was no difference in rates of non-union (16% vs 11%, p=0.5), amputation (0% vs. 3%; p=0.44) or deep bone infection (7% vs. 11%; p=0.74) between the elderly and under-65s, respectively.

### Free flap cohort

A total of 54 patients received free flap reconstruction between February 2012 and August 2019 with a mean age of 39 (± 17) years (Table 4). Forty-eight patients were aged <65 years and 6 patients were aged ≥65 years at the time of free flap reconstruction; 67% of patients received soft tissue coverage with an anterolateral thigh (ALT) flap. The incidence of medical comorbidities (hypertension and diabetes mellitus) was comparatively higher in the patients aged ≥65, see Table 4. Microvascular venous thrombosis occurred in 4 patients (7%), all of whom were aged <65 years. Partial flap necrosis occurred in 8 patients (15%), all of whom were aged <65 years and achieved definitive soft tissue coverage with debridement, flap advancement and split skin grafting to residual bare areas.

**Table 4:** Comparative injury-specific and outcome data for all patients who received free flap reconstruction stratified by age between years 2012–2019.

Gender	Aged <65 (n= 48)	Aged ≥65 (n= 6)
Male	41 (85%)	5 (83%)
Female	7 (15%)	1 (17%)
**Mechanism**		
RTA	36 (75%)	4 (67%)
Fall	7 (15%)	2 (33%)
Chronic wound	2 (4%)	-
Crush injury	1 (2%)	-
Penetrating trauma	1 (2%)	-
Bomb blast	1 (2%)	-
**Gustilo-Anderson grade**		
IIIb	46 (96%)	6 (100%)
IIIc	2 (4%)	-
**Flap type**		
ALT	33 (69%)	3 (50%)
LD	4 (8%)	-
RFF	5 (11%)	-
MSAP	4 (8%)	3 (50%)
Gracillis	1 (2%)	-
DIEP	1 (2%)	-
**Number of venous anastomoses**		
1	17 (35%)	2 (33%)
2	28 (59%)	4 (67%)
3	3 (6%)	-
**Medical co-morbidities**		
Hypertension	1 (2%)	3 (50%)
Diabetes mellitus	1 (2%)	3 (50%)

Abbreviations: ALT: Anterolateral thigh, DIEP: Deep inferior epigastric perforator, LD: Latissimus dorsi, MSAP: Medial sural artery perforator, RFF: Radial forearm flap, RTA: Road traffic accident.

## Discussion

During the study period, approximately 1 in 5 patients presenting with an open tibia/fibula fracture were aged ≥65 years. Following debridement, the grade of injury in terms of Gustilo-Anderson classification was broadly similar in younger vs. elderly groups, despite the different energy mechanisms. Elderly patients had a significantly longer time to debridement and trended towards worse compliance with BOAST guidelines for time to debridement and soft tissue coverage. Overall, the type of bony stabilisation and soft tissue coverage was similar across age groups. There was no difference in rates of non-union or amputation, despite the elderly having many more co-morbidities. Our data shows that elderly and young patients are sustaining similar injuries in terms of soft tissue grading, despite different mechanisms. It therefore follows that current guidelines are still relevant in the elderly population, although modifications to improve outcomes is still possible.

Our findings are limited by the single centre, retrospective nature of this study and smaller sample size. Similarly, the outcome data are limited by the degree of follow up and documentation, and many patients from out of the area could not be included due to a lack of follow-up data. In our institution, we do not routinely document the fragility scores of elderly patients being admitted for major trauma so it was not available for data capture. In order to improve the evidence base, we recommend that frailty scores be recorded in future studies and incorporated into the updated BOAST guidelines as part of the orthogeriatric review. The development of a core outcome set for open tibia/fibula fractures would standardise outcome reporting on this topic and facilitate evidence synthesis.^20^ By including consecutive patients, we aimed to reduce bias. We excluded patients who died before definitive limb reconstruction because we wished to focus our study on patients surgically fit for reconstruction, and this exclusion may bias our results towards fitter elderly patients who were able to survive the initial insult of the trauma. While this study is not designed to identify small differences in management between groups, the strength of this study lies in its ability to rapidly identify important differences, as demonstrated by the length of hospital stay and this knowledge can readily inform guidelines and practice.

The bimodal age and gender distribution of open tibia/fibula fractures in this study are consistent with previous UK data.^21^, ^22^, ^23^ This study demonstrated a similar soft tissue insult in terms of Gustilo-Anderson classification despite the difference in mechanism. This observation may be explained by skin fragility and systemic frailty,^22^^,^^24^ allowing a greater degree of tissue destruction despite lower forces involved. Physiologically, however, higher energy injuries have a larger zone of injury and thus are more likely to be associated with vascular complications^25^; this is reflected in our data showing vascular complications only in the younger (18–65 years) age group. It follows that older patients should be considered for broadly similar operative management as younger patients with similar soft tissue injury grades, despite comorbidities such as atherosclerosis, as long as they are surgically fit.

This study did not demonstrate a longer time to antibiotic administration in the elderly, which is in contrast to previous studies.^26^ Unlike operative interventions which may require anaesthetic assessment, there is no reason why elderly patients should experience a delay in antibiotic administration and we suggest that units monitor their compliance locally.

Elderly patients had a significantly longer time to debridement. This is in keeping with previous data^26^ and may be due to a greater proportion of lower energy injuries and the need for comprehensive anaesthetic review.^27^, ^28^, ^29^ Peri-operative optimisation is likely to be more important for elderly patients to reduce the risks of surgery and anaesthesia, and the delay seems justified. We suggest that current targets for time to debridement be reviewed for elderly patients, with a view to either implementing pathways for prioritised anaesthetic assessment or changing targets to facilitate optimisation. Previous research has shown that elderly trauma patients are less likely to be transported to trauma centres (‘undertriage’) and there may be an element of age bias which delays other aspects of care, such as antibiotic administration and timely debridement.^7^^,^^30^ Although difficult to prove, unconscious age bias could affect care. Appropriate training on the recognition and treatment of elderly trauma should be provided for all members of the multidisciplinary team, including prehospital providers.^30^

In comparison with previous data from this unit,^17^^,^^31^ we report similar proportions of definitive skeletal fixation methods (external fixation, intramedullary nailing); however, lower rates of free flap coverage and amputation which could reflect the lower proportion of severe injuries (Gustilo-Anderson classification IIIb or greater) in this study and inter-observer variability in injury severity classification systems.^32^ Soft tissue injury severity classification was graded by the operating surgeon immediately after the first debridement. Interobserver variability in Gustilo-Anderson grading by the operating surgeon was more difficult to control in our study due to the retrospective nature. Our data on method of soft tissue coverage and initial skeletal fixation are consistent with previous studies in elderly cohorts with similar injury severities.^23^^,^^33^ Some surgeons may be reluctant to perform free flap reconstruction in the elderly, owing to concerns regarding comorbidities and flap survival. In the extended free flap cohort, microvascular venous thrombosis and partial flap necrosis were only reported in the 18–65 years age group, despite more comorbidities in the elderly, including diabetes mellitus. The total free flap failure rate we have reported is similar to that in INTELLECT, a large prospective study of open extremity fractures (n= 2,694) including 62 hospitals across 16 countries (personal communication). Although we reported a low number of free flaps in elderly patients, the available data do not support higher free flap complications in those aged ≥65 years. Those aged ≥65 years who were surgically fit for free flap reconstruction appeared to do well, despite the presence of risk factors for poor healing, highlighting the importance of early anaesthetic input. Other options for soft tissue coverage in those unsuitable for prolonged surgery include VAC dressings.^34^ Biodegradable Temporising Matrix is also being increasingly used for traumatic soft tissue defects.^35^

Despite a higher frequency of comorbidities, there was no difference in frequency of non-union, amputation or deep bone infection between age groups, contrary to previous reports.^26^ These findings are supported by Clement et al., who demonstrated that non-union was not associated with age in their cohort of open tibial fractures, but rather with higher energy mechanisms.^36^ Compared to previous data we report lower rates of amputation overall; this may relate to the lower proportion of Gustilo-Anderson grade 3 injuries in our cohort.^23^^,^^26^ Additionally, despite similar operative interventions across both age groups, elderly patients had a significantly longer time to discharge. Length of stay in both age groups was comparable with previous reports.^23^^,^^37^^,^^38^ Longer recovery times in elderly patients are expected due to reduced physiological reserves,^39^ and co-morbidities may also lengthen their stay. It seems reasonable to give equal access to limb reconstruction based on the above, and future work into enhanced care pathways that could accelerate the rehabilitation of these patients in a cost-effective manner should be undertaken, as has been the case for elderly neck of femur fractures.^40^^,^^41^

## Conclusion

Open tibia/fibula fractures in elderly patients are usually low energy but sustain a similarly graded soft tissue injury to younger patients with high-energy injuries. This similarity in injury profile allows current guidelines to maintain relevance in this population, despite them not being written for the elderly. The operative management of open tibia/fibula fractures in elderly and younger patients is also similar, as were key outcomes such as the risk of non-union or amputation. Comorbidities do not seem to impact non-union or amputation, however, may be one of the factors involved with the longer length of hospital stay in the elderly. This study provides evidence that reconstruction is a realistic aim for elderly patients who are fit for surgery. We recommend that future guidelines prioritise the peri-operative optimisation of elderly patients over the exact timing of debridement, aiming for faster post-operative recovery and discharge for this population.
